# Evidence-Based Public Health

**Published:** 2004-03-15

**Affiliations:** Steps to a HealthierUS, National Center for Chronic Disease Prevention and Health Promotion, Centers for Disease Control and Prevention, Atlanta, Ga

In a relatively short amount of time, the term “evidence-based public health” has flooded dialogues on program planning, implementation, and evaluation. What is evidence-based public health? Abigail Adams reminded us that “[w]e have too many high sounding words, and too few actions that correspond with them” (1). In *Evidence-Based Public Health*, Brownson and colleagues provide not only a precise definition of a complex term but also a stepwise framework for decision making toward improved public health practice.

The authors order the text according to a 6-step process for enhancing evidence-based decision making in public health: 1) develop an initial statement of the issue; 2) quantify the issue; 3) search the scientific literature and organize the information; 4) develop and prioritize program options; 5) develop an action plan and implement interventions; and 6) evaluate the program or policy. With every step in the process, the authors provide resources for immediate use, including Wide-ranging OnLine Data for Epidemiologic Research (WONDER), a Centers for Disease Control and Prevention (CDC) program; the Community Health Status Indicators Project; the *Annual Review of Public Health*; evidence-based information on health care outcomes, quality, cost, use, and access via the Agency for Healthcare Research and Quality (AHRQ); the Guide to Community Preventive Services; the Models that Work Campaign to identify and promote innovative community-based models; the Planned Approach to Community Health (PATCH); PRECEDE-PROCEED; and the CDC Working Group on Evaluation.

*Evidence-Based Public Health* was prepared for 4 main user groups: public health practitioners, policy makers, researchers, and key stakeholders, including the public. The text should be considered necessary reading in schools of public health; the authors artfully marry science and practice with accessible case examples throughout. The combination of practical steps and supporting resources serves as a foundation for decision making in public health as a tangible product of lessons learned via traditional research, the ongoing translation of diverse sources of evidence, and reflective practice. Without question, the authors demystify “evidence-based public health” and delve into intimately related concepts, including the role and varying quality of “best practices” in public health.

Yet, with an increasing emphasis on more integrated, community-based approaches to chronic disease prevention and health promotion, the authors leave room for others to undertake a much-needed discussion of the back-and-forth relationship between emerging or “promising practices” and how this information might expand on existing evidence to inform public health practice now and in the future. As we strive to identify important leverage points for improving community and health outcomes, how will lessons learned in the front lines of public health practice infiltrate that which constitutes “good” evidence? Do community-based practitioners have the resources necessary to evaluate programs and disseminate key findings? Have we created ample pathways for informing evidence-based decision making in public health?

The book lends itself to a follow-up discussion of community-based participatory research as a possible strategy for enhancing the evidence base relevant to program development to address a wide range of existing and emerging health disparities. Moreover, *Evidence-Based Public Health* highlights the necessity of continued investment in research syntheses, as well as strategies of dissemination that take into account the real-world challenges faced by practitioners in a climate of uncertain resources and increasing calls for accountability to new and diverse stakeholders. To this end, the authors surely set the stage for rich dialogue on a host of issues critical to advancing chronic disease prevention and health promotion in bold new directions.
